# Factors influencing French community pharmacists’ willingness to participate in research projects: a mixed method study

**DOI:** 10.1186/s12875-023-02163-w

**Published:** 2023-11-03

**Authors:** Arthur Piraux, Morgane Angibaud, Pierre Nizet, Dominique Navas, Claire Sallenave-Namont, Sonia Prot-Labarthe, Jean-François Huon

**Affiliations:** 1 Department of Pharmacy, Univ Angers, CHU Angers, Angers, F-49000 France; 2 Primary Care Federative Department, Faculty of Medicine, University of Nantes, Nantes, F-44000 France; 3 UMR INSERM 1246 SPHERE “methodS in Patient-centered Outcomes and HEalth ResEarch, Nantes Université, Université de Tours, Nantes, France; 4 Nantes Université, CHU Nantes, Pharmacie, Nantes, F-44000 France; 5 UR2160 ISOMer - UFR de Sciences Pharmaceutiques et biologiques, Université de Nantes, Nantes, F- 44035 France; 6 Nantes Université, CHU Nantes, Université Paris Cité, Inserm, ECEVE, F- 75010 Pharmacie, Paris, France

**Keywords:** Community pharmacy research, Pharmacists, Research, Primary healthcare, Barriers, Facilitators

## Abstract

**Background:**

French community pharmacists are facing an increasing demand to provide a wider range of services to meet the needs of the population. These new missions must be evaluated by primary care research studies. This study aims to explore the factors that influence French community pharmacists’ willingness to participate in research projects.

**Methods:**

A mixed-method design was adopted for this study, comprising an initial quantitative online survey followed by semi-directed interviews. The investigation was conducted at two French faculties of pharmacy, Angers and Nantes, involving students in their 6th and final year of pharmacy education, and their community pharmacist tutors. The survey items were based on a study by Saini et al. and participants responded using five-point Likert scales. The semi-directed interviews were conducted after the quantitative analysis, only with volunteer and already graduated community pharmacists.

**Results:**

A total of 131 people participated in the quantitative analysis, comprising 75 students and 56 pharmacists. Pharmacists and students agreed on the significance of two key aspects: the research must possess a clear and meaningful purpose, and researchers must keep the pharmacists informed about the study’s results. Among the 27 proposed items, only three showed significantly different results between students and pharmacists. Moreover, 11 semi-structured interviews were conducted. Research in the community pharmacy domain is relatively new for many pharmacists. Despite limited training, their willingness to participate is contingent on being actively involved from the outset, receiving appropriate support and training. However, the research should be seamlessly integrated into their daily practice, without being too time-consuming and administratively burdensome. Time constraints emerged as the main obstacle, along with concerns about the availability of human resources. Pharmacists expressed strong motivation driven by the research topic’s relevance, and its potential impact on patients or the profession. While financial compensation is desirable, it did not appear to be the main criterion for participation in a study.

**Conclusions:**

French pharmacists are willing to participate in research projects to improve patient care and develop the profession. Research teams must guide and involve from the project’s inception.

**Supplementary Information:**

The online version contains supplementary material available at 10.1186/s12875-023-02163-w.

## Background

Community pharmacists play a critical role as primary health care providers, offering easy accessibility and availability to patients. They are often sought out as the first point of contact for healthcare advice, and also serve at the final stage of the treatment process by providing medications [1, 2]. However, challenges in accessing traditional health care services and lack of physicians were one of the drivers for the expansion of the community pharmacist’s competencies [3]. The COVID-19 pandemic further accelerated the process, particularly in the context of screening and vaccination efforts [4–6]. Evaluation of these new services would improve our understanding of the impact of pharmacists on these practices and their evolving role [7, 8]. Furthermore, it is worth noting that, although these studies involve community pharmacists, they are often conducted by academic researchers, with pharmacies serving primarily as data collection sites or recruitment centers.

Several countries have already examined the role of the community pharmacist in research and the factors that influence their participation. The underlying assumption is that the involving community pharmacists in practice-based research can significantly enhance pharmacy practice [9]. Notably, a recent study conducted in Lebanon reported that more than two-thirds of pharmacists (68.5%) expressed an interest in participating in research projects [10]. Similarly, a study from the Netherlands emphasized the importance of community pharmacy research, with over 80% of pharmacists acknowledging that research provides evidence-based insights into the actions of community pharmacists [11]. In contrast, in the United Kingdom, involvement in research projects can be made mandatory, resulting in nearly 90% of pharmacists responding to a survey having already participated in research programs. However, only 56.7% of respondents considered research to be of significant importance to their practice [12].

Despite the significant expansion of the roles of community pharmacists in the past few years, no study has yet been conducted in France to explore their willingness to engage in research project. The introduction of various missions, such as pharmaceutical interviews (2012), medication assessments (2018), flu vaccination (2019), monitoring of patients undergoing oral chemotherapy (2020), protocol dispensing (2021), and others vaccinations (2022) [13–17] highlights the relevance and necessity of analyzing the French context. Hence, this is why our study aims to explore the factors influencing the willingness of French community pharmacists to participate in research projects.

## Method

This study adopts an observational and prospective research design. A mixed method was used to explore the opinion of community pharmacists and final year community pharmacy students from the *Pays de la Loire* region, France. The research is reported in compliance with the STROBE checklist for the quantitative part, and the COREQ checklist for the qualitative part [18, 19]. This bicentric study was conducted in the Faculty of Pharmacy of Angers and the Faculty of Pharmacy of Nantes, in accordance with French regulations and has received approval from the Institutional Review Board of the *Groupe Nantais d’Ethique dans le Domaine de la Santé* (IRB #21-12-028). The study was carried out by experienced community and hospital pharmacists possessing extensive knowledge of both community pharmacy practice and research methodologies. Their affiliation with the university system provided a seamless connection with the participating students and their tutors.

### Population

Community pharmaceutical studies in France extend over a six year period. During their last year, students undertake a six-month internship in a pharmacy under the guidance of a pharmacist tutor. For this study, a total of eighty students (49 students in Angers and 31 in Nantes), along with their community pharmacist tutors, were eligible and offered the opportunity to participate. As a first step, all students were invited to complete an online questionnaire. Afterwards, the tutor pharmacists hosting these students were also invited to take part in the study. Additionally, other pharmacists working in these pharmacies had the option to participate in the survey. As a result, the participating pharmacists encompassed various practice settings, including both urban and rural areas, reflecting diverse modes of practice. The survey invitation was disseminated during a meeting, and the link to the questionnaire was sent by e-mail. Each university contacted its own students through a mailing list, and completion of the questionnaire was considered as an expression of consent.

At the end of the questionnaire, pharmacists were invited to partake in semi-structured interviews. Those interested were requested to leave their e-mail address, allowing the research team to contact them. Prior to commencing the interviews, each participant provided informed oral consent and agreed to the recording of the interview.

### Data collection

The quantitative part was carried out using an online survey on the Microsoft Forms® platform (Additional file 1). This survey consisted of three sections: socio-demographic and community pharmacy data, previous participation in research projects and the pharmacist’s opinion on research. The latter section was adapted from the study by Saini et al. which identified factors influencing Australian community pharmacists’ willingness to participate in research projects [20]. Each proposal from the reference study was translated and adapted to align with the French health system by a research team comprising two academic and community pharmacists, one academic pharmacist, and four academic and hospital pharmacists. The questionnaire and interview guide were pilot tested by two different community pharmacists. The questionnaire employed five-point Likert scales to obtain responses (1 point = strongly disagree to 5 points = strongly agree). Mailing lists were used to distribute the survey, and data collection occurred between February and April 2022.

For the qualitative component, pharmacists interesting in participating in semi-directed interviews were initially contacted via e-mail to schedule an appointment. The interviews were then conducted either in person, *via* videoconference, or telephone, depending on the pharmacy’s location and the availability of the participants. Alongside note-taking, the interviews were recorded and transcribed using the NVIVO V.12.7.0 software (QRS International Pty. Ltd., Doncaster, Victoria, Australia). Recruitment for interviews continued until data saturation, signifying that two consecutive interviews no longer revealed new themes relevant to the research question. The semi-structured interviews were conducted from July to September 2022 by several researchers trained in this interview approach (DN, MA). A semi-structured interview guide comprised of four sections (Additional file 2) was designed.

The first part of the guide aimed to explore the pharmacist’s representations of research and their personal understanding of the term. The second section explored pharmacists’ perceptions of “primary care research”, with inquiries concerning their thoughts, expectations and potential barriers if invited to participate in such projects. The third part of the guide explored the pharmacist’s motivations and levers influencing their engagement in research initiatives. Finally, the fourth part allowed for a reflective review of the interview and the opportunity to synthesize the gathered insights.

### Data analysis

For the quantitative study, qualitative data were presented using frequencies and percentages. Quantitative variables were analyzed using means and standard deviation. Differences between students and pharmacists were compared using Student’s t-tests. A *p*-value of < 0.05 was considered statistically different, while a *p*-value of < 0.001 was considered highly significant.

Regarding the qualitative study, after checking the transcripts, the data was analyzed independently by two authors (MA and SPL), adopting a thematic approach [21]. This approach treats data collection and analysis as simultaneous stages, with constant back and forth interactions between the field and the interpretation. Thus, the four stages of this analysis (immersion in the data, coding, creation of categories and identification of themes) were conducted by authors who compared their findings until they reached a common interpretation of the verbatims collected. Triangulation was employed to improve the quality of data collection, coding and analysis. Thus, to prevent subjectivity or over-interpretation of the data, the coding and analysis issues were systematically discussed with the research team.

## Results

### Characteristics of the population

A total of 131 individuals participated in this study (Table 1). The majority of participants was students (n = 75), accounting for over 93.0% of the total student population, and 57.5% of pharmacists. Approximately two thirds of the participants were female, comprising 68.0% of students and 60.7% of pharmacists. The majority of students were under 30 years old. In contrast, pharmacists who responded to the survey represented various age groups.

Of the pharmacist respondents, only 4 (7.1%) had been previously involved in research projects. Out of these, half were pharmacy owners, and the other half were employee pharmacists. Only one student had prior experience participating in a research project, and that involvement occurred during his time as a hospital student.

**Table 1:** Characteristics of the population (n = 131)

Variable	Value	Students n = 75	Pharmacists n = 56
**Sex**	Women	51 (68.0%)	34 (60.7%)
**Age ** **(year)**	[20–30[	74 (98.7%)	6 (10.7%)
	[30–40[	1 (1.3%)	10 (17.9%)
	[40–50[	0	15 (26.8%)
	[50–60[	0	14 (25.0%)
	≥ 60	0	11 (19.6%)
**Faculty**	Angers	45 (60.0%)	*NA*
	Nantes	30 (40.0%)	*NA*
**Work experience** **(year)**	< 10	*NA*	7 (12.5%)
	[10–20[	*NA*	18 (32.1%)
	[20–30[	*NA*	16 (28.6%)
	≥ 30	*NA*	15 (18.8%)

NA: not applicable. Proportion were calculated among students and pharmacists

### Quantitative results

The results of the 27 items are presented in Table 2. Both pharmacists and students reached a consensus on the two proposals they considered most and least important. Firstly, they agreed that research should have a clear and meaningful purpose (item 19) and researchers should inform pharmacists of the results of the study (item 21). On the other hand, promoting pharmacist involvement in research as an attractive factor for the pharmacy (item 8) and active participation in developing research project methods (item 4) received the lowest ratings from the respondents.

Only three items showed significantly different results between students and pharmacists. Pharmacists expressed higher interest in being involved in research (item 1) and exhibited greater confidence in their ability to conduct research compared to students (item 14). In contrast, students expressed more doubts regarding patient recruitment than pharmacists (item 23).

**Table 2:** Mean score for factors influencing community pharmacists’ and pharmacy students’ willingness to participate in a research project

Item	Proposal	Pharmacists	Students	p-*value*
**1**	**I would like the pharmacy I work in to be actively involved in research**	**3.71**	**3.25**	**< 0.01**
2	I would be involved in research if I have a special interest in the specific topic being investigated	4.04	4.25	0.13
3	I would like to be actively involved in developing new ideas for future research	3.02	3.13	0.48
4	I would like to be actively involved in developing methods of research projects	2.71	2.47	0.17
5	I would like to be actively involved in research that I thought would benefit my patients	4.09	4.17	0.60
6	Being actively involved in research would give me a chance to do something out of the ordinary in my pharmacy	3.75	3.68	0.67
7	Being actively involved in research would improve community perceptions of the pharmacy	3.64	3.49	0.44
8	Promoting my involvement in research with the university would help my business	2.7	2.63	0.68
9	The commitment to quality and training is important to me.	4.34	4.21	0.33
10	I believe research should generate income for the pharmacy	4.18	4.15	0.83
11	Pharmacists should be paid for participating in research study	4.14	4.09	0.74
12	Patients participating should receive incentives other than possible improvement in health	3.25	3.37	0.47
13	I think it is important for research to occur within community pharmacy settings	3.63	3.69	0.64
**14**	**I am quite confident of being able to conduct research in my day-to-day practice**	**3.29**	**2.84**	**0.01**
15	If I were involved in research, I would like frequent contact with researchers	3.71	3.89	0.22
16	During research I would like to contact more experienced pharmacists	3.96	4.16	0.21
17	I would like extensive training in how to organize the research activity	3.66	3.89	0.20
18	During research I would like to be involved with other professionals	4.23	4.29	0.59
19	It would be important that there is a clear and meaningful goal to the research	4.66	4.59	0.47
20	It would be important that I feel the results were directly applicable to my pharmacy	4.14	4.27	0.35
21	It would be important that researchers inform me of the results of the study	4.45	4.56	0.30
22	Most projects seem difficult because they require pharmacy restructuring	3.43	3.69	0.09
**23**	**I feel that it is difficult to interest patients in research participation**	**3.16**	**3.61**	**0.01**
24	I feel time constraints restrict me from participating in research projects	4.04	4.23	0.24
25	I feel that the lack of trained staff is a reason that conducting research is difficult in my pharmacy	3.63	3.55	0.69
26	I feel that other healthcare professionals do not value pharmacy-based research	3.59	3.72	0.39
27	I feel I need extensive training before undertaking research	4.2	4.16	0.80

Responses were scored as follows: 1 = strongly disagree, 2 = disagree, 3 = neither agree nor disagree, 4 = agree, 5 = strongly agree

Bold items indicate significant results

### Qualitative results

#### Participants characteristics

Data saturation occurred after conducted the 11th interview. Table 3 presents the main characteristics of pharmacists participating in these interviews. The gender and age of the pharmacists and the typology of pharmacies were varied, and all categories were represented. Of the 5 departments in the *Pays de la Loire* region, two were over-represented, namely 44 and 49, which are university towns.

**Table 3:** Characteristics of pharmacists participating in semi-directed interviews

Interviews		Pharmacists		Pharmacies	
Number	Duration (*min:sec*)	Gender	Age (*years*)	Typology	Faculty
#1	20:33	Woman	31–40	Urban	Nantes
#2	37:06	Woman	51–60	Urban	Angers
#3	47:24	Man	51–60	Urban	Nantes
#4	36:44	Woman	≥ 60	Urban	Nantes
#5	35:45	Man	41–61	Rural	Nantes
#6	44:01	Man	41–61	Rural	Angers
#7	25:40	Man	≥ 60	Rural	Angers
#8	29:28	Men	51–60	Urban	Angers
#9	26:05	Woman	20–30	Rural	Angers
#10	24:12	Woman	31–40	Urban	Nantes
#11	27:21	Man	31–40	Rural	Nantes

Three main categories emerged from the data analysis: the lack of knowledge of community pharmacist about research projects, the existence of barriers and facilitators for community pharmacists, and the proposition of several recommendations to improve research projects including community pharmacists.

#### Primary care research: a new concept for community pharmacists

First, the ‘research’ term was perceived as abstract by most of pharmacists. The research objectives were perceived as encompassing both broad and specific aspects. Research was described as having a theoretical foundation and should solve a problem.

*‘For me, research is very theoretical. It’s vague but it’s interesting, it allows us to move forward.’ #1*.

Community pharmacists expressed a limited sense of involvement in research, primarily due to their perception of lacking the necessary skills to participate. They did not consider the pharmacy setting to be ideal to practice research, especially when it involved fundamental or experimental research.

*‘This is not the place! We’re in community pharmacy, so we certainly don’t do experimental research.’ #2*.

One of the main difficulties for defining research was the lack of knowledge about this practice. Several pharmacists openly admitted uncertainty and sought for clarification. Frequently, research was confused with experiments conducted for new missions.

*‘Then there were projects, such as pharmacies in Angers that offered “smoking cessation initiations”, that sort of thing. But I don’t see this as research. There are pharmacies, for example, that are starting to work on urinary tract infections. I don’t see that as research either.‘ #11*.

However, despite the difficulty in defining the scope of pharmaceutical research, some saw the pharmacy as a suitable place to conduct research. Some pharmacists expressed frustration as they unable to fully participate in such projects, especially after six years in university. One pharmacist even went so far as to say that he felt “under-used”.

Despite the perceived lack of research in community pharmacy felt by the pharmacists, several research projects were mentioned, in which some participants had actively contributed. For instance, one pharmacist participated in a study on medication reviews, another in a study on diabetes, and several contributed to students’ theses. It is worth noted that, in their opinion, these activities were not considered research per se, but projects, innovation, or experimentation. Although many of the pharmacists interviewed were unfamiliar with the concept of research, or had not participated in it, several ideas emerged. Numerous pharmacists referred to research on the new missions proposed for pharmacists such as prevention, vaccination, and pharmaceutical interviews, among others. Specific pathologies, including diabetes, cancer, and age-related conditions, were also identified as research areas of interest. Several pharmacists mentioned that research results had influenced changes in their daily practices, emphasizing the practical and patient-beneficial nature they wanted the research to have.

*‘Medication reviews, all the tests we can perform like pharyngitis, COVID now, diabetes, and everything from blood pressure to vaccination. All these new missions are part of the new missions we have.’ #10*.

Through these examples, pharmacists have expressed an interest in participating in primary care research that promises to be beneficial. First, there were benefits to the patient, particularly improved patient care. A better understanding of treatment, a reduction of drug-related problems, and improvement in quality of life were mentioned. Subsequently, they perceive personal and professional gains, primarily in terms of optimizing their daily practices. Research would help improve practices and positively impact health outcomes. It would provide a new perspective on their practice, and thus enable them to improve. Many pharmacists saw it as a source of professional fulfillment. Finally, some pharmacists had in mind the benefits for the healthcare system. By conducting research within community pharmacy, pharmacists believed it could yield cost savings for society. They argued that it would reduce the cost of care, avoid inappropriate utilization of emergency departments, and ultimately increase the system’s efficiency.

*‘It’s about advancing our practices and advancing healthcare in general.’ #1*.

*‘A profession that studies, that reflects, that produces on itself. I think this is a very positive image.’ #5*.

*‘The aim would be for the person, the patient, the client to find a benefit, otherwise there is no point.’ #6*.

*‘ If I may speak for myself, professional fulfilment.’ #12*

#### Barriers and facilitators identified for implementation of primary care research

The main issue reported by the majority of pharmacists was the lack of available time. They expressed that their schedules were already packed with various tasks and responsibilities, leaving little room for additional commitments. While they were aware that involvement in research would require time allocation, they were reluctant to devote personal time, which was already very much taken up by their professional practice. Some of them suggested the possibility of organizing meetings during the lunch break or in the evenings, but others dismissed this idea.

*‘A community pharmacy may not have the time to set up the research.’ #11*.

While some pharmacists might manage to find time to participate in research, the issue of finding suitable replacement during their absence was raised. The opening hours of a community pharmacy were contingent on the effective presence of a pharmacist, leading to the challenge of reconciling research commitments with maintaining uninterrupted service to patients. Again, some participants mentioned the difficulty of recruiting, finding assistants or replacements. The matter of remuneration and additional costs associated with arranging for replacements further complicated the situation. One pharmacist emphasized the need for an adequate number of assistants to support research activities. In other words, the desire to engage in research projects seemed to be hampered by the difficulty of finding the time to do so.

*‘But even I, when I am absent, a pharmacy technician does not replace me. You need a pharmacist, a pharmacist costs 30 euros an hour. And then you have to find one. And then, for half a day, you can’t find anyone!’ #3*.

In addition to the lack of time, pharmacists also reported the organizational difficulties associated with establishing pharmacy research, particularly the administrative burden and procedural complexities. They expressed a preference for a streamlined and straightforward research process that could seamlessly integrate into their daily routine, without becoming burdensome. In practice, the research investigation should have an appropriate duration to enable them to implement it within their practical limitations. On one hand, the research period should not be overly condensed, affording them sufficient time to execute it effectively. On the other hand, it should not be too long to prevent loss of motivation and falling behind schedule.

*‘It shouldn’t be too complicated, because if there are procedures… We are fed up with all these procedures. And when it is too complicated or too heavy, it is discouraging.’ #2*.

To address these barriers, pharmacists have put forward several policy levers. First of all, the research topic should be of interest for them. Pharmacists emphasized that their motivation and level of engagement were important for them, and would be significantly heightened if the subject matter directly related to patient care or was relevant to pharmacy practice. In other words, research topics that hold practical relevance and the potential to drive meaningful changes in their professional practice were perceived as more enticing and likely to elicit greater participation.

*‘If it’s not a topic I’m particularly interested in, I’m not going to make any effort.’ #4*.

*‘If they are projects that really aim to improve [practices] and are this time implemented at the end of the project, yes, and on the contrary, that is all beneficial.’ #10*.

They were also interested in participating in a research project with the rest of the team, in priority the other pharmacists, but also the pharmacy technicians. They added that senior students would be very helpful, especially during their pharmacy internship.

*‘What would be nice for community pharmacists is to motivate the assistants. I think it’s interesting to involve the pharmacy technicians as well.’ #7*.

#### Recommendations to develop primary care research by community pharmacists

Pharmacists offered a variety of recommendations aimed at fostering the development of community pharmacy research. Regarding the implementation of a new study, they wish to be involved right from the inception of the study, rather than solicited solely during the experimental phase. They felt that they were in the best position to give their opinion on studies directly relevant to their practice, enabling them to avoid potential pitfalls.

*‘Being part of the people who think about how it can be implemented. Yes, that could be interesting because we know the daily life of the community pharmacist, which is perhaps not the case for all hospital pharmacists.’ #3*.

*‘I will be more comfortable implementing [the study] rather than participating in the research and methodology development stage.’ #12*.

However, participation in research would enhance the value of pharmacists’ knowledge and skills. This implied having a solid foundation, particularly in terms of research methodology. Many pharmacists admitted to having limited experience in developing rigorous methodologies. Consequently, several expressed a desire for access to appropriate training, as well as personalized support, such as guidance from a tutor. They believed that regular check-ins would help sustain motivation and keep them on track throughout the research process.

*‘At least a training at the beginning, I think that we know what it takes, what you want, what we can give you.’ #8*.

*‘Well, afterwards, we need a tutor to set yourself up, since you are often a beginner in this field, so you need a tutor.’ #9*.

In addition, a direct exchange between the research team leading the project and the pharmacists themselves was deemed crucial. They suggested the establishment of regular meetings, either in person or virtual, or communication through emails. Regarding meetings, they recommended having multiple sessions and being informed well in advance so that they can organize themselves.

*‘Afterwards, nothing prevents you from contacting everyone by e-mail and proposing a meeting, even by video or other means, but I am less convinced of the effectiveness.’ #5*.

In their view, the university was considered the most favorable entity to provide research opportunities to pharmacists, primarily due to their shared academic backgrounds. In particular, they gave it a scientific guarantee. However, they also highlighted the significance of the representative bodies, such as the French chamber of pharmacist, the unions, pharmacists’ groups, and the Regional Unions of Health Professionals support, which were recognized as potential avenues to facilitate research engagement.

*‘The Regional Unions of Health Professionals is already carrying a lot of projects. And of course the universities. The universities must take on the role of scientific guarantor.’ #7*.

The objective of pharmacists was to simplify processes without adding complexity to the daily routines of the pharmacy teams. Remuneration was also discussed. While pharmacists did not wish to conduct research at a financial loss, especially by employing a pharmacy assistant, for many of them remuneration was not the primary motivation. Instead, many insisted on the necessity of an interesting subject before considering any form of compensation.

*‘If it’s paid, if there’s remuneration involved, that’s the icing on the cake, but it’s not the primary motivation.’ #7*.

## Discussion

This observational study demonstrates that French community pharmacists exhibit a strong desire to actively engage in research, and that they have many propositions to encourage pharmacists to participate in research projects, but also to help researchers include them in their projects. Conducting the qualitative study after the quantitative study provided valuable insights and a deeper understanding of this phenomenon. It provided a more comprehensive understanding of our research topic, offered explanatory insights, and brought a human element to the findings about community pharmacists participation in research.

### Knowledge and interest of French community pharmacists to participate in research projects

Although direct comparison of our results with the study by Saini et al. is not feasible, a similar trend can be observed [20]. For example, a clear and meaningful research objective, coupled with effective communication of the study results to community pharmacists, emerged as key factors influencing their willingness to participate in the research. Pharmacists displayed a certain reluctance to actively participate in the entire process but expressed a willingness to be consulted when necessary. A prominent restricting factor for pharmacist involvement in research, as reported in previous studies, was the lack of time [10].

Similar to their foreign colleagues, French community pharmacists hold distinct views regarding the significance and the relevance of research. Research is frequently associated with the experimentation of new missions. Despite the theoretical aspect of this term, respondents recognized its contribution to their professional practice, their culture, but also the improvement of patient care. Findings from a comparable study conducted in the United Kingdom by Crilly et al. also corroborate some of our study results [12], in particular the importance of research for the profession’s future (60% of pharmacists), the development of novel services (87%), and the need to stay informed about research relevant to pharmacy practice (85%). Moreover, a recent study revealed a positive correlation between a favorable attitude towards practice-based research and the utilization of the best current scientific evidence [9]. This finding can be attributed to pharmacists’ interest in research, especially when the topic aligns with their personal interests. The motivation to participate and undergo training becomes more straightforward in such cases.

In Australia, the pharmacist profession is undergoing changes, research and development playing an important role in supporting these transformations. Similarly, community pharmacists in France aim to use research projects to improve their practice, and develop new pharmacy services [22]. This perception is shared among Swedish community pharmacists, where research projects are viewed as means to promote community pharmacies to patients, and contribute to the development of new services [23]. However, community pharmacists often lack experience and feel uncomfortable with conducting such research due to perceived knowledge gaps. The acquisition of these new skills requires training, but also support from research teams [9, 23]. Despite this, there are obstacles to the development of community-based pharmaceutical research, but some levers are identified to foster its growth and success.

### Barriers and facilitators to implement primary care research

The main constraint identified is the limited availability of pharmacists to participate in a research project. This was reported by the vast majority of pharmacists surveyed, and aligns with existing literature [24–26]. French pharmacists attributed this lack of time to difficulties in recruiting both pharmacist assistants and technicians. It should be noted that this study was conducted shortly after the COVID-19 pandemic, with significantly increased pharmacists’ workload, leading some to leave the profession [27]. Consequently, the remaining pharmacists had to handle increased responsibilities with fewer staff.

Replacing the participating pharmacist during research further compounds the time constraint. Indeed, the absence of a pharmacist, whether for training, a home visit or other reasons, must be compensated by the presence of another qualified pharmacist to keep the pharmacy open. To alleviate this constraint, the pharmacists interviewed suggested holding meetings during lunchtime or evening, and providing additional staff support. This observation is consistent with the Kentucky Agency for Healthcare Research and Quality findings, which emphasized the importance of providing human resources, with approximately one in two pharmacists viewing the provision of a network research assistant as “very important” [26].

Overall, pharmacists require a genuine interest in research to encourage them investing time and resources. Respondents mentioned apprehensions about bureaucratic hurdles and organizational difficulties within the pharmacy setting. A Lebanese study reported similar barriers in terms of lack of time, personal resources, and support [10]. Consistent with the article published by Saini et al., prior participation in research has been show to increase interest in engaging in further research endeavors [20].

### Benefits and supports of community pharmacy research

The benefits of community pharmacist participation in research are multiple, benefiting both patients and pharmacists themselves [28–31]. Patients are willing to participate in research, particularly when the outcomes focus on their well-being and needs (patient-centered outcomes research). Considering the important relationship that already exists between patients and community pharmacists, the pharmacy setting appears to be conducive to including patients in research projects [32]. After all, patients are the main subjects of study. Secondly, it will benefit the entire profession allowing for the evaluation and potential modification of certain practices. Finally, their participation will be of direct interest to research teams, especially universities that oversee these projects [33, 34].

To encourage pharmacists to participate in research projects, investigating teams are advised to involve community pharmacists from the outset, not solely for recruitment purpose. While pharmacists may not seem to be interested in the construction of the methodology or the protocol in detail, they do desire a clear understanding of the research endeavor they are engaging in. This finding may be one of the explanations for the score observed for item 4 (active participation in the development of the research project) which was the lowest in the questionnaire. Pharmacists expressed the desire to be informed from the initial stages of the research project to discuss its, the relevance of research questions, and the type of intervention that would be implemented, in order for research to be aligned with their practice. Nonetheless, they do not envisage significant involvement to the extent of drafting a research protocol as they are less interested in the purely technical aspects of methodology. This aligns with the current trend of involving stakeholders from the inception of research projects, including them in the design process [35–37]. A need for training has been identified and will enable pharmacists to limit deviations from the research protocol [38]. They also request regular communication with the research teams, and access to support such as a tutor in case of need. As pharmacists typically work alongside assistants, students at the end of their studies and technicians, it is suggested to include them in research projects as well [39]. The quantitative analysis showed only three significant differences between the two groups interviewed, likely attributable to inexperience of the students being probably the main reason of these differences.

Institutional support for pharmacy teams is important. While academics are not the only promoters of research, pharmacists perceive their involvement as a reassuring guarantee. Certain countries, such as Denmark, Australia and the United States, have created research networks dedicated to community pharmacists [39–41]. These networks not only strengthens pharmacy practice and research, but also foster connections between pharmacists and researchers. The more pharmacists are involved in research projects, the more representative the evidence base will be. The results obtained from such research will help in modifying or developing new practices [42].

Financial compensation is almost always requested by participating pharmacists in research endeavors. It is often seen as an incentive to their involvement [11, 38]. However, French pharmacists prioritize the value of research and its impact on patient care over financial remuneration. They believe that money should not be the primary motivation for their participation in research. As some studies point out, pharmacists are primarily seeking compensation for time dedicated to research rather than purely monetary rewards. This distinction should be clear to researchers who want the support and engagement of community pharmacists in their research initiatives [39].

### Limitations

This study has some limitations that were addressed to the best extent possible. Firstly, the questionnaire required some modifications to suit the French context, including translation. Although external validation of translation was not feasible, all team members involved contributed until a consensus was reached (a backward and forward translation process could have been performed). Secondly, the study was conducted in a single French region, which could lead to a representativeness bias. This bias was mitigated by the participation of two faculties of pharmacy in the region as well as by the numerous respondents from different settings. However, further research would be needed to develop guidelines for research teams to facilitate the engagement of community pharmacists in research projects.

## Conclusion

Community pharmacy practices are undergoing changes, and the involvement of pharmacists in research is among the notable transformations. Primary care research supports the development and evaluation of new clinical missions. This study provided some answers about the willingness of French community pharmacists to participate in research projects.

Pharmacists are ready to embrace this new facet of their work if they receive proper support. This support should be built upon three fundamental pillars: training, organization, and recognition. Research should be integrated into their daily practice, not impose excessive time constraints, and address topics of interest. Projects supported by universities or professional organizations serve as motivating factors and guarantee research quality.

### Electronic supplementary material

Below is the link to the electronic supplementary material.

Supplementary Material 1

Supplementary Material 2

Supplementary Material 3

## Data Availability

All data generated or analyzed during this study are included in this published article and its supplementary information files.
