# Supplementary material for: Factors influencing French community pharmacists’ willingness to participate in research projects: a mixed method study
Source: BMC Prim Care. 2023 Nov 3;24:229. doi: 10.1186/s12875-023-02163-w (PMC10623853; doi:10.1186/s12875-023-02163-w)
Supplement: Supplementary file 1 — Supplementary Material 1 [file 12875_2023_2163_MOESM1_ESM.docx]

Additional file 2: Interview guide

"Hello, I am XXX. Thank you for receiving me for this interview. The idea is to find out what you think about primary care research as a community pharmacist. I am going to ask you some questions that I have on my interview guide to explore your representations of research in pharmacy. If you wish to interrupt the interview, you may do so at any time.

This interview will be recorded, to be transcribed into a text file. The recording will then be deleted. It is important to know that it is anonymous, i.e., nobody else will know that it is you who took part in the interview, as you will be given an ID to preserve your anonymity. The interview will last about 30 minutes and once the interview is over, we can debrief what you thought of it, or even answer your questions if you have any.

One last thing, you might see me taking some notes, it's just so that I don't forget some elements that I would have questioned and that I would like to come back to later. So, if you don't mind, we can start, and I will start the recording".

1. Tell me what research means to you.
2. What does community pharmacy research mean to you?
3. What do you think about research in primary care/community pharmacy?
4. What would you think if you were offered to participate in a research project?
5. What suggestions would you make for community pharmacists to participate more in research projects?
6. Is there anything I have forgotten? Is there anything you would like to discuss around this topic?
