# Supplementary material for: Factors influencing French community pharmacists’ willingness to participate in research projects: a mixed method study
Source: BMC Prim Care. 2023 Nov 3;24:229. doi: 10.1186/s12875-023-02163-w (PMC10623853; doi:10.1186/s12875-023-02163-w)
Supplement: Supplementary file 2 — Supplementary Material 2 [file 12875_2023_2163_MOESM2_ESM.pdf]

# Additional file 1: Questionnaire

Research in community pharmacies is developing with more and more ambitious projects such as the evaluation of the impact of medication assessment on hospitalizations, or the evaluation of the management of simple cystitis by the pharmacist. Through this questionnaire, we ask you about your involvement and your vision of research in pharmacy.

This questionnaire must be completed first by the student in the 6th year, then by the training supervisor with the student. It can also be completed by other pharmacists in the team.

The questionnaire lasts 10 minutes.

## Your situation

1. Your are:
    a. A women
    b. A men
2. You are
    a. Between 20 and 30 years old
    b. Between 31 and 40 years old
    c. Between 41 and 50 years old
    d. Between 51 and 60 years old
    e. Over 60 years old
3. You are
    a. Pharmacy student
    b. Pharmacist assistant
    c. Pharmacist

## Your education

1. You are dependent on
    a. The University of Angers
    b. The University of Nantes

## Your experience

1. You have been working in a pharmacy for
    a. Less than 5 years
    b. Between 5 and 10 years
    c. Between 11 and 20 years
    d. Between 21 and 30 years
    e. More than 30 years
2. Your pharmacy is located
    a. In a rural area
    b. In a neighbourhood
    c. In town
    d. In a shopping center
    e. Other: …

## Previous participation in research projects

1. Have you ever participated in any pharmacy research projects?

   As a reminder, participation in thesis questionnaires is not included in the term "research projects".

a. Yes

b. No

2. What projects have you participated in?

   a. …

3. Who was the leader(s) of the research project(s)?

   a. …

4. How were you approached to participate?

   a. …

## Research for you

For each of the following statements, please indicate your level of agreement.

| | Strongly disagree | Disagree | Neither agree or disagree | Agree | Strongly agree |
|---|---|---|---|---|---|
| I would like the pharmacy I work in to be actively involved in research | | | | | |
| I would be involved in research if I had a special interest in the specific topic being investigated | | | | | |
| I would like to be actively involved in developing new ideas for future research | | | | | |

| | | | | | |
|---|---|---|---|---|---|
| I would like to be actively involved in developing methods and materials for the day-to-day running of research projects | | | | | |
| I would like to be actively involved in research that I thought would benefit my customers | | | | | |
| Being actively involved in research would give me a chance to do something out of the ordinary in my pharmacy | | | | | |
| Being actively involved in research would improve community perceptions of the pharmacy | | | | | |
| Advertising my involvement in research with the university would help my business | | | | | |
| Continuous professional education/continuous quality improvement points for involvement is important to me | | | | | |
| Pharmacists should be paid for participating | | | | | |
| Patients participating should receive incentives other than possible improvement in health | | | | | |
| I think it is important for research to occur within community pharmacy settings | | | | | |

| | | | | | |
|---|---|---|---|---|---|
| I am quite confident of being able to conduct research in my day-to-day practice | | | | | |
| If I were involved in research I would like frequent contact with researchers | | | | | |
| During research I would like to contact more experienced pharmacists for monitoring | | | | | |
| I would like extensive training in how to organise the research activity | | | | | |
| During research I would like to be involved with other professionals | | | | | |
| It would be important that there was a clear and meaningful goal to the research | | | | | |
| It would be important that I felt the results were directly applicable to my pharmacy | | | | | |
| It would be important that researchers informed me of the results of the study | | | | | |
| Most projects seem difficult because they require pharmacy restructuring | | | | | |
| I feel that it is difficult to interest patients in research participation | | | | | |
| I feel time constraints restrict me from participating in research projects | | | | | |

| | | | | | |
|---|---|---|---|---|---|
| I feel that the lack of trained staff is a reason that conducting research is difficult in my pharmacy | | | | | |
| I feel that other healthcare professionals do not value pharmacy-based research | | | | | |
| I feel I need extensive training before undertaking research | | | | | |

## Availability for an interview

We would like to complete the study with interviews to explore the determinants of pharmacists' participation in research. These will be semi-structured individual interviews of approximately 30 minutes in length and will be voice recorded.

1. If you are available for an interview, please provide your email address and we will contact you afterwards.
    a. …
