# Supplementary material for: Factors influencing French community pharmacists’ willingness to participate in research projects: a mixed method study
Source: BMC Prim Care. 2023 Nov 3;24:229. doi: 10.1186/s12875-023-02163-w (PMC10623853; doi:10.1186/s12875-023-02163-w)
Supplement: Supplementary file 3 — Supplementary Material 3 [file 12875_2023_2163_MOESM3_ESM.pdf]

Enquête RecOFF

La recherche en officine se développe avec des projets de plus en plus ambitieux comme l'évaluation de l'impact du bilan de médication sur les hospitalisations, ou l'évaluation de la prise en charge de la cystite simple par le pharmacien. À travers ce questionnaire, nous vous interrogeons sur votre implication et sur votre vision de la recherche en officine.

Ce questionnaire doit être complété dans un premier temps par l'étudiant en 6ème année, puis par le maître de stage avec l'étudiant. Il peut être également complété par d'autres pharmaciens de l'équipe.
La durée du questionnaire est de 10 minutes.

* Obligatoire

Votre situation

1. Vous êtes *

- ○ Une femme
- ○ Un homme

2. Vous avez *

- ◯ Entre 20 et 30 ans
- ◯ Entre 31 et 40 ans
- ◯ Entre 41 et 50 ans
- ◯ Entre 51 et 60 ans
- ◯ Plus de 60 ans

3. Vous êtes *

- ◯ Etudiant.e en pharmacie
- ◯ Pharmacien adjoint
- ◯ Pharmacien titulaire

Votre formation

4. Vous dépendez de *

- ◯ La faculté de pharmacie d'Angers
- ◯ La faculté de pharmacie de Nantes

Votre expérience

5. Vous exercez en officine depuis *

- ◯ Moins de 5 ans
- ◯ Entre 5 et 10 ans
- ◯ Entre 11 et 20 ans
- ◯ Entre 21 et 30 ans
- ◯ Plus de 30 ans

6. Votre pharmacie est située *

- ◯ En milieu rural
- ◯ En milieu urbain

Votre pharmacie

7. Vous exercez en officine depuis *

- ◯ Moins de 5 ans
- ◯ Entre 5 et 10 ans
- ◯ Entre 11 et 20 ans
- ◯ Entre 21 et 30 ans
- ◯ Plus de 30 ans

8. Votre pharmacie est située *

- ◯ En milieu rural
- ◯ En quartier
- ◯ En ville
- ◯ Entre centre commercial
- ◯ Autre

9. Le nombre de pharmaciens équivalent temps plein (ETP) dans l'officine est de *

10. Le chiffre d'affaire de la pharmacie (en euros) est *

- Inférieur à 500.000
- Entre 500.000 et 1 million
- Entre 1 million et 2 millions
- Entre 2 millions et 3 millions
- Plus de 3 millions

Participation antérieure à des projets de recherche

11. Avez-vous déjà participé à des projets de recherche en officine ? *

Pour rappel, la participation à des questionnaires de thèses n'est pas inclus dans le terme "projets de recherche".

◯ Oui

◯ Non

12. Quels sont les projets auxquels vous avez participé ? *

13. Qui étai(en)t le(s) porteur(s) de ce(s) projet(s) de recherche ? *

14. Comment avez-vous été sollicité pour participer ? *

La recherche pour vous

15. Pour chacune des affirmations suivantes, veuillez donner votre degré d'accord. *

	Pas du tout d'accord	Pas d'accord	Ni d'accord, ni en désaccord	D'accord	Tout à fait d'accord
Je souhaiterais que la pharmacie dans laquelle je travaille participe activement à la recherche.	○	○	○	○	○
Je participerais à la recherche si j'ai un intérêt particulier pour le sujet spécifique étudié.	○	○	○	○	○
J'aimerais participer activement à l'élaboration de nouvelles idées pour de futures recherches.	○	○	○	○	○
J'aimerais participer activement à l'élaboration de	○	○	○	○	○

méthodologie des projets de recherche.					
J'aimerais participer activement à des recherches qui, selon moi, profiteraient à mes patients.	○	○	○	○	○
Participer activement à une recherche me donnerait l'occasion de faire quelque chose qui sort de l'ordinaire dans ma pharmacie.	○	○	○	○	○
Participer activement à la recherche améliorerait la perception de la pharmacie par la population.	○	○	○	○	○
Promouvoir ma participation à la recherche universitaire favoriserait mon officine au niveau économique.	○	○	○	○	○
L'engagement dans la démarche qualité et [illegible]	○	○	○	○	○

dans la formation sont importants pour moi.	◯	◯	◯	◯	◯

16. Pour chacune des affirmations suivantes, veuillez donner votre degré d'accord. *

	Pas du tout d'accord	Pas d'accord	Ni d'accord, ni en désaccord	D'accord	Tout à fait d'accord
Je pense que la pharmacie devrait être indemnisée pour la participation à la recherche.	◯	◯	◯	◯	◯
Les pharmaciens devraient être rémunérés pour leur participation.	◯	◯	◯	◯	◯
Les patients qui participent devraient recevoir des incitations autres qu'une éventuelle amélioration de leur santé.	◯	◯	◯	◯	◯
Je pense qu'il est important que la recherche se déroule au sein de la pharmacie d’officine.	◯	◯	◯	◯	◯

Je suis assez confiant dans ma capacité à mener des recherches dans ma pratique quotidienne.	○	○	○	○	○
Si je participais à une recherche, je souhaiterais avoir des contacts fréquents avec les chercheurs.	○	○	○	○	○
Pendant la recherche, j'aimerais pouvoir contacter des pharmaciens plus expérimentés.	○	○	○	○	○
Je souhaiterais recevoir une formation approfondie sur l'organisation de la recherche.	○	○	○	○	○

17. Pour chacune des affirmations suivantes, veuillez donner votre degré d'accord. *

	Pas du tout d'accord	Pas d'accord	Ni d'accord, ni en désaccord	D'accord	Tout à fait d'accord
Pendant la recherche [illegible]					

recherche, j'aimerais être impliqué avec d'autres professionnels.	○	○	○	○	○
Il serait important que la recherche ait un objectif clair et significatif.	○	○	○	○	○
Il serait important que les résultats soient directement applicables à ma pharmacie.	○	○	○	○	○
Il serait important que les chercheurs m'informent des résultats de l'étude.	○	○	○	○	○
La plupart des projets me semblent difficiles car ils nécessitent une réorganisation de la pharmacie.	○	○	○	○	○
J'estime qu'il est difficile d'intéresser les patients à participer à la recherche.	○	○	○	○	○

Je pense que les [illegible]

contraintes de temps m'empêchent de participer à des projets de recherche.	○	○	○	○	○
J'estime que le manque de personnel qualifié est une raison pour laquelle il est difficile de mener des recherches dans ma pharmacie.	○	○	○	○	○
J'ai l'impression que les autres professionnels de la santé ne valorisent pas la recherche en pharmacie.	○	○	○	○	○
J'estime avoir besoin d'une formation approfondie avant de conduire des recherches	○	○	○	○	○

Disponibilité pour un entretien

Nous souhaitons compléter l'étude par des entretiens pour explorer les déterminants de la participation à la recherche par les pharmaciens. Ce sont des entretiens individuels semi-dirigés d'environ 30 minutes enregistrés vocalement.

18. Si vous êtes disponible pour un entretien, merci d'indiquer votre adresse mail et nous prendrons contact avec vous par la suite

Ce contenu n'a pas été créé ni n'est approuvé par Microsoft. Les données que vous soumettez sont envoyées au propriétaire du formulaire.

Microsoft Forms
